# Supplementary figures and images for: CT radiomics for differentiating fat poor angiomyolipoma from clear cell renal cell carcinoma: Systematic review and meta-analysis
Source: PLoS One. 2023 Jul 27;18(7):e0287299. doi: 10.1371/journal.pone.0287299 (PMC10374097; doi:10.1371/journal.pone.0287299)

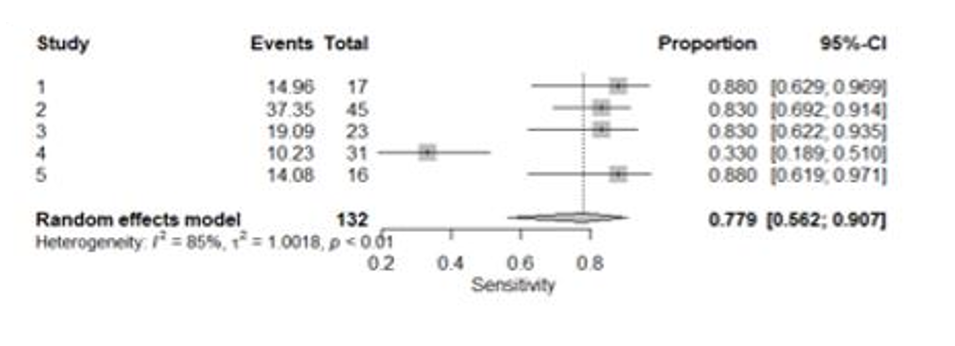

Supplement: S1 Fig — (TIF) [file pone.0287299.s002.tif]

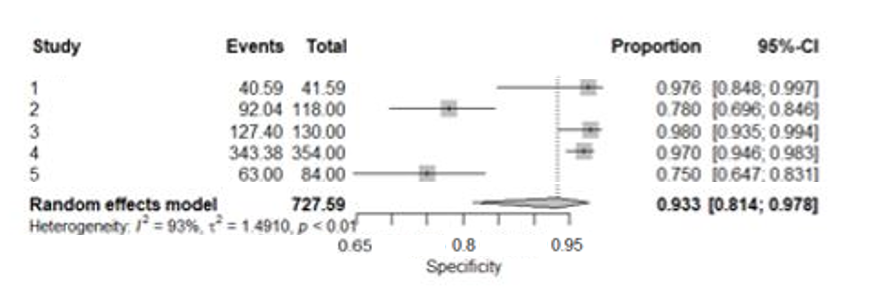

Supplement: S2 Fig — (TIF) [file pone.0287299.s003.tif]

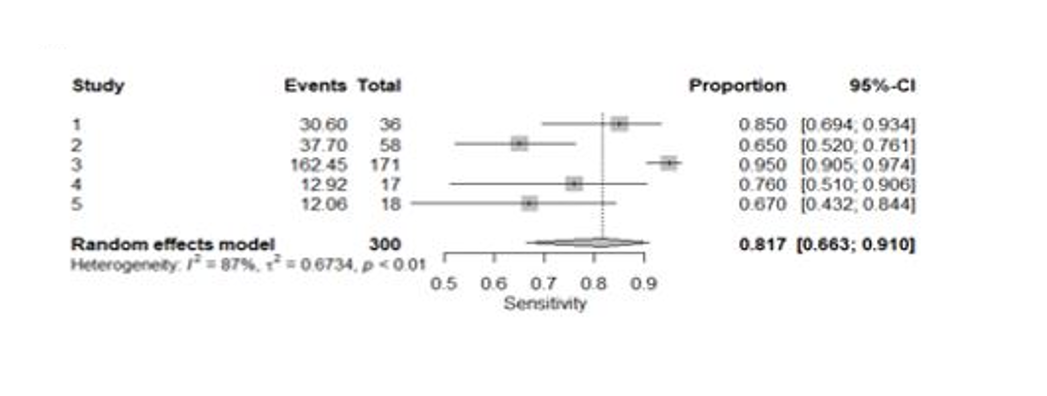

Supplement: S3 Fig — (TIF) [file pone.0287299.s004.tif]

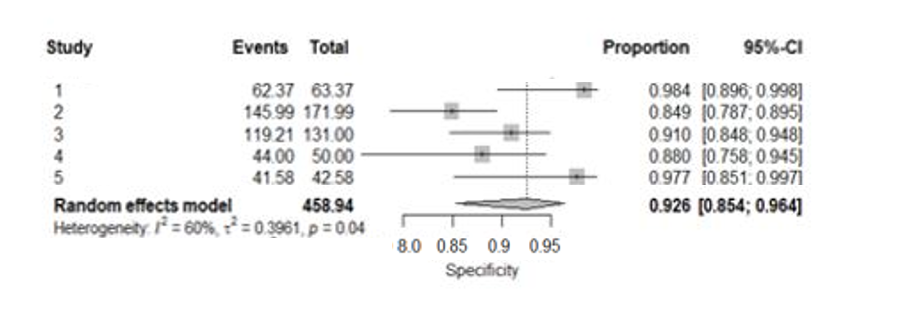

Supplement: S4 Fig — (TIF) [file pone.0287299.s005.tif]
