# Supplementary material for: CT radiomics for differentiating fat poor angiomyolipoma from clear cell renal cell carcinoma: Systematic review and meta-analysis
Source: PLoS One. 2023 Jul 27;18(7):e0287299. doi: 10.1371/journal.pone.0287299 (PMC10374097; doi:10.1371/journal.pone.0287299)
Supplement: S1 Table — (DOCX) [file pone.0287299.s006.docx]

**Supplementary table 1.** Pooled sensitivity, specificity, and odds ratio for differentiating between AMLs without visible fat (fp-AML) from renal cell carcinomas (group 1) and clear cell renal cell carcinomas (group 2)

| Groups | Sensitivity(95% CI) | Heterogenicity% | Specificity(95% CI) | Heterogenicity % | Odds ratio(95% CI) | Heterogenicity % |
| --- | --- | --- | --- | --- | --- | --- |
| Best performance (Group1) | 0.779 [0.562; 0.907] | 85 | 0.933 [0.814; 0.978] | 93 | 41.8223 [12.7880; 136.7768] | 68 |
| Best performance (Group2) | 0.817 [0.663; 0.910] | 87 | 0.926 [0.854; 0.964] | 60 | 58.6729 [15.1248; 227.6075] | 87 |
