# Supplementary material for: CT radiomics for differentiating fat poor angiomyolipoma from clear cell renal cell carcinoma: Systematic review and meta-analysis
Source: PLoS One. 2023 Jul 27;18(7):e0287299. doi: 10.1371/journal.pone.0287299 (PMC10374097; doi:10.1371/journal.pone.0287299)
Supplement: S2 Table — (DOCX) [file pone.0287299.s007.docx]

**Supplementary table 2.** Pooled sensitivity, specificity, and odds ratio for differentiating between AMLs without visible fat (fp-AML) from clear cell renal cell carcinomas (group 2) based on different CT phases

| CT phases (for group 2) | Sensitivity(95% CI) | Heterogenicity % | Specificity(95% CI) | Heterogenicity % | Odds ratio(95% CI) | Heterogenicity % |
| --- | --- | --- | --- | --- | --- | --- |
| Unenhanced CT Scan | 0.858 [0.742; 0.927] | 45 | 0.886 [0.819; 0.930] | 0 | 43.0819 [14.1095; 131.5459] | 30 |
| Corticomedullary phase | 0.755 [0.628; 0.850] | 30 | 0.882 [0.814; 0.927] | 0 | 22.6278 [10.0476; 50.9589] | 0 |
| Nephrographic phase | 0.781 [0.656; 0.870] | 0 | 0.832 [0.734; 0.899] | 0 | 18.0281 [6.7723; 47.9911] | 32 |
