# Supplementary material for: CT radiomics for differentiating fat poor angiomyolipoma from clear cell renal cell carcinoma: Systematic review and meta-analysis
Source: PLoS One. 2023 Jul 27;18(7):e0287299. doi: 10.1371/journal.pone.0287299 (PMC10374097; doi:10.1371/journal.pone.0287299)
Supplement: Attachment — Submitted filename: Response to reviewers.docx [file pone.0287299.s008.docx]

Dear Dr. Zahid Mehmood,

Thank you so much indeed for your consideration regarding our manuscript entitled “**CT Radiomics for Differentiating Fat Poor Angiomyolipoma from Clear Cell Renal Cell Carcinoma: Systematic Review and Meta-Analysis**”. Also, we are deeply thankful to the reviewer whose comments improved the quality of our paper.

**Editor Comments:**

Please revise your paper according to the following suggested changes;
1. The authors need to re-write the abstract again to show the objective of this paper clearly.
2. Introduction section needs to be finished with summary and the contribution should be pointed out.
3. The proposed approach each stage is not discussed in details, nor each stage is interlink appropriately and the discussion is very poor. Please revise it carefully.
4. The author also needs to make a clear conclusion/novelty at the end of each sub-section of methodology.
5. In result section, the authors require to present clear details about the data analysis.
6. The following recent studies on should be discussed in related work;

- Data hiding technique in steganography for information security using number theory, 2019
- MedDeblur: Medical Image Deblurring with Residual Dense Spatial-Asymmetric Attention, 2022
- Stress Estimation Model for the Sustainable Health of Cancer Patients, 2022
- Publishing and interlinking covid-19 data using linked open data principles: toward effective healthcare planning and decision-making, 2022
- Image authenticity detection using DWT and circular block-based LTrP features, 2019

**Response:** Thank you for your comments. Could you please confirm whether these comments are associated with this manuscript since we noticed that the articles you recommended to discuss in the manuscript are not pertinent to our subject matter.

**Reviewers' comments:**

**Reviewer #1:**

1. A common radiologic dilemma is still not avoided for CT imaging to differentiate fat-poor angiomyolipomas (fp-AMLs) from clear cell renal cell carcinomas (ccRCCs) due to the limitation of density of CT images. CT radiomics may improve to present the renal mass characteristics on basis of CT images, and many studies proved that it is a good tool in detecting fp-AMLs from RCCs. This article about the systematic Review and Meta-Analysis of CT Radiomics for Differentiating fp-AMLs from ccRCCs will be benifit to acknowledge the role of CT radiomics.
2. Radiomics is still being a hot topic because it is a promising and powerful addition to the diagnostic armamentarium, and the limitations is evident in the design and execution of the studies and these limit its translation into clinical practice.

**Comment 1.** The distinctive characteristics of CT radiomics are not deep analyzed and not widely acknowleged. This article did not present the different signs of CT radiomics in detail for RCCs and fp-AMLs, and the cited articles are small.

**Response 1:** Thanks for your comment. We acknowledged your concern regarding “analyzing and discussing distinctive characteristics and signs of CT radiomics” by adding one paragraph in discussion and one paragraph in the limitation.

Regarding your comment on the limited number of cited articles, we made a conscious effort to concentrate on studies that met our inclusion criteria from 2011 to 2022, as these were the articles that had undergone the three stages of screening. We were not able to consider any recent studies from June 2022 to March 2023 as they had not been evaluated for inclusion criteria and our manuscript has been under review at PLOS ONE for the past nine months.

Please let us know if there are any other changes required.

Thank you so much for your time and consideration.

Best regards,

Ashkan Malayeri
